# Effect of prone position on respiratory parameters, intubation and death rate in COVID-19 patients: systematic review and meta-analysis

**DOI:** 10.1038/s41598-021-93739-y

**Published:** 2021-07-13

**Authors:** Fatemeh Behesht Aeen, Reza Pakzad, Mohammad Goudarzi Rad, Fatemeh Abdi, Farzaneh Zaheri, Narges Mirzadeh

**Affiliations:** 1 Student Research Committee, School of Nursing and Midwifery, Shiraz University of Medical Sciences, Shiraz, Iran; 2 Faculty of Health, Ilam University of Medical Sciences, Ilam, Iran; 3 Master of Critical Care Nursing, Tehran University of Medical Sciences, Tehran, Iran; 4 School of Nursing and Midwifery, Alborz University of Medical Sciences, Karaj, Iran; 5 Non-communicable Diseases Research Center, Alborz University of Medical Sciences, Karaj, Iran; 6 Midwifery Department, Kurdistan University of Medical Sciences, Sanandaj, Iran; 7 Department of Midwifery, School of Nursing and Midwifery, Bam University of Medical Sciences, Bam, Iran

**Keywords:** Diseases, Health care, Medical research

## Abstract

Prone position (PP) is known to improve oxygenation and reduce mortality in COVID-19 patients. This systematic review and meta-analysis aimed to determine the effects of PP on respiratory parameters and outcomes. PubMed, EMBASE, ProQuest, SCOPUS, Web of Sciences, Cochrane library, and Google Scholar were searched up to 1st January 2021. Twenty-eight studies were included. The Cochran's Q-test and I^2^ statistic were assessed heterogeneity, the random-effects model was estimated the pooled mean difference (PMD), and a meta-regression method has utilized the factors affecting heterogeneity between studies. PMD with 95% confidence interval (CI) of PaO_2_/FIO_2_ Ratio in before–after design, quasi-experimental design and in overall was 55.74, 56.38, and 56.20 mmHg. These values for Spo_2_ (Sao_2_) were 3.38, 17.03, and 7.58. PP in COVID-19 patients lead to significantly decrease of the Paco_2_ (PMD: − 8.69; 95% CI − 14.69 to − 2.69 mmHg) but significantly increase the PaO_2_ (PMD: 37.74; 95% CI 7.16–68.33 mmHg). PP has no significant effect on the respiratory rate. Based on meta-regression, the study design has a significant effect on the heterogeneity of Spo_2_ (Sao_2_) (Coefficient: 12.80; p < 0.001). No significant associations were observed for other respiratory parameters with sample size and study design. The pooled estimate for death rate and intubation rates were 19.03 (8.19–32.61) and 30.68 (21.39–40.75). The prone positioning was associated with improved oxygenation parameters and reduced mortality and intubation rate in COVID-19 related respiratory failure.

## Introduction

Recently a new virus called coronavirus 2019 (COVID-19) is spreading all around the world^1,2^ and caused a global pandemic with increasing incidence, mortality, and medical resource consumption which impose enormous socio-economic burdens^3,4^. COVID-19 disease ranges from mild respiratory tract illness to severe progressive pneumonia, primarily manifesting as acute respiratory distress syndrome (ARDS) requiring admission to the intensive care unit (ICU)^4^. ARDS occurs in 20–41% of patients^5^. The mortality rate among ARDS patients is high and has been reported to be between 30 and 40%^6,7^. Higher mortality of COVID-19 patients may be related to higher incidences of barotrauma and ventilator-induced lung injury (VILI)^8^. The COVID-19 pandemic presented a unique challenge for the health care systems. The shortage of resources is one of these problems that pandemic imposed, include human resources, ICU beds, and mechanical ventilators^9^. In the absence of effective therapies for COVID-19, the implementation of supportive care is essential^10^. Prone positioning is one of these interventions for patients with severe ARDS, which could improve oxygenation and has a survival benefit^11^ and also could improve outcomes in COVID-19 patients. It has been suggested as the standard of care in international guidelines^12^. Proning can reduce ventral-dorsal trans-pulmonary pressure differences and lung compression by the heart and diaphragm, resulting in lung perfusion improvement. Proning has been demonstrated to improve lung compliance and lung recruitability and reduce VILI incidence^8^. Studies of prone ventilation in COVID-19 ARDS patients have shown improvement in the ratio of the partial pressure of arterial oxygen (PaO_2_) to the fraction of inspired oxygen (FiO_2_) (PaO_2_/FiO_2_)^8^ by 35 mmHg^13^. Prone positioning may play a role in reducing systemic inflammation by increasing alveolar fluid drainage. Inflammatory responses related to ARDS or secondary to VILI may be attributed with pulmonary and extra-pulmonary organ dysfunction and strategies to reduce inflammation may lead to increased survival^8^. Prone positioning also increases chest wall elastance and amplifies active expiration during coughing^14^. Studies report that prone positioning reduced 28-day and 90-day mortality rates^15,16^ and accelerated the time for extubation^15^. The World Health Organization (WHO) recommends its use for periods of 12–16 h/day^17–19^.

Correct selection of patients and applying the accurate treatment protocol for prone positioning are crucial to its efficacy^6^. Special precautions are required for placing and monitoring a patient in the prone position^20^. Intubated patients in prone positioning are at risk, such as accidental removal of the tracheal tube, pressure ulcer, facial edema, gastroesophageal reflux, and other problems. Overall, it seems that correct patient selection, timely initiation, and duration of patient’s placement in this position can all affect the effectiveness of this intervention^6^. Considering that COVID-19 is a novel disease that caused many difficulties and due to lack of sufficient evidence, the need to assess the effects of prone positioning as a supportive care in hypoxemic patients is necessary, so we conducted this systematic review and meta-analysis to determine the effects of prone position on respiratory parameters and outcomes of COVID-19 patients.

## Materials and methods

In accordance with the Preferred Reporting Items for Systematic Reviews and Meta-Analyses (PRISMA) guidelines for designing and implementing systematic review studies, the following steps were taken: a systematic literature search, organization of documents for the review, abstracting and quality assessment of each study, synthesizing data, and writing the report^21^. The protocol of the study was registered in the International Prospective Register Of Systematic Reviews (PROSPERO) at the National Institute For Health Research. Registration number in PROSPERO is CRD42021257619.

### Search strategy

According to the PICO framework, the systematic literature search was conducted on PubMed, EMBASE, ProQuest, SCOPUS, Web of Sciences, Cochrane library, and Google Scholar databases. MeSH Keywords were connected with AND, OR and NOT prone position and respiratory parameters, and their suggested entry terms were the main keywords in the search strategy.

1. 'Coronavirus Disease 2019 [Title/Abstract], OR 'COVID-19' [Title/Abstract], OR 'Coronavirus' [Title/Abstract], OR 'SARS-cov-2' [Title/Abstract], OR 'Sever acute respiratory syndrome coronavirus-2' [Title/Abstract], '2019-nCov' [Title/Abstract], OR 'SARS-Cov' [Title/Abstract]
2. 'Prone' [Title/Abstract], OR 'Prone position' [Title/Abstract]
3. 'Oxygenation' [Title/Abstract], OR 'Cell Respiration' [Title/Abstract], OR 'Cell Respirations' [Title/Abstract]
4. 'Respiratory Distress Syndrome' [Title/Abstract], OR 'Acute respiratory distress syndrome' [Title/Abstract] OR 'Hypoxemic' [Title/Abstract], OR 'Respiratory Insufficiency' [Title/Abstract], OR 'Dyspnea' [Title/Abstract]
5. 1 AND 2
6. 1 AND 2 AND 3
7. 2 AND 3AND 4
8. 1 AND 2 AND 3 AND 4

Population, Intervention, Comparators, Outcomes (PICO) criteria for this study includes (P): patients with COVID-19. (I): prone position. (C): no intervention, (O): respiratory parameters and outcome.

### Inclusion and exclusion criteria

#### Type of studies

Studies including quasi-experimental and before–after designs were included if the effects of prone position on respiratory parameters were reported as an outcome. Also, studies met the inclusion criteria if they were published until 1st January 2021. There was no language filtering. The case report, case series, reviews, and studies with incomplete data were excluded**.**

#### Type of participants

The studies were selected if participants were patients with Reverse transcription polymerase chain reaction (RT-PCR) confirmed test or if imaging findings showed evidences of COVID-19, patients with COVID-19 need oxygenation (face mask, nasal cannula, invasive mechanical ventilation, non-invasive mechanical ventilation). Pregnant women, patients who have prone positioning contraindication such as skeletal fractures were excluded.

#### Type of intervention

Patients were instructed to stay in the prone position based on the proning protocol of each study for at least 30–60 min and then return to the supine position. Standard prone position was considered for 16 h/day (some studies considered the duration of prone position ≥ 3–4 h, or until the patient is uncomfortable). The average time of prolonged sessions was considered up to 36 h. However, in one study, a 5-min protocol was used. Respiratory parameters were measured three times in most studies (before positioning, during prone position, and after prone position).

#### Type of outcomes measure

The primary outcome was the respiratory parameters and respiratory status. The secondary outcomes were death rate and intubation rates (Supplementary 1).

### Study selection

**Figure 1:**
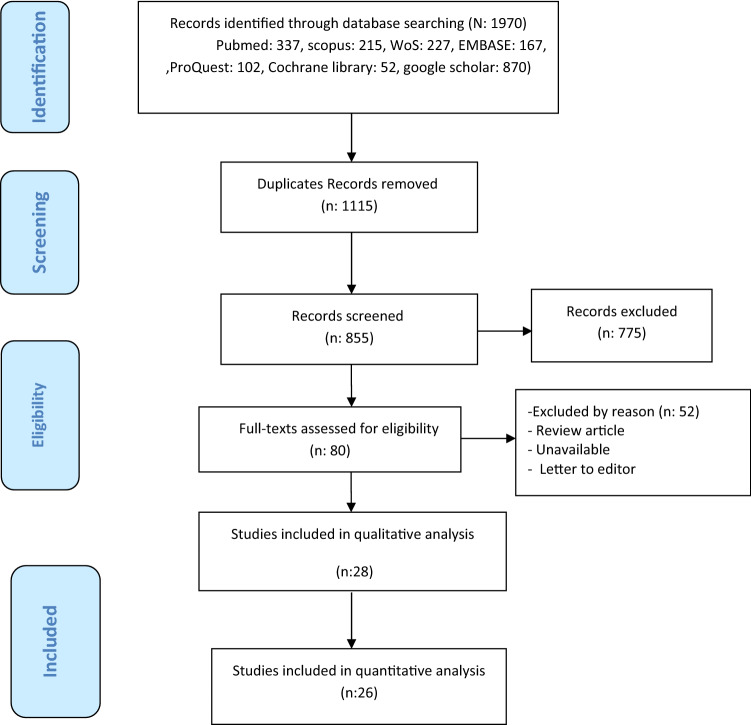
PRISMA diagram for searching resources.

Two authors independently evaluated the eligibility of these articles, and any disagreements were resolved by consensus. Several articles were excluded due to being irrelevant or duplicated. Finally, 28 full-text articles were included in the systematic review and 26 articles in the meta-analysis (Fig. 1).

### Risk of bias and quality assessment

The methodological quality of the included studies in this review was conducted by the Mixed Methods Appraisal Tool (MMAT). The quality assessment was conducted independently by two authors. The MMAT was developed to appraise different empirical studies categorized into five categories: qualitative, randomized controlled trial, nonrandomized, quantitative descriptive, and mixed methods studies^22^. This tool consists of five items for each category, each of which could be marked as Yes, No, or cannot tell. Based on the scoring system, score one is assigned to Yes and score 0 to all other answers. In other words, the total score would be the percentage of affirmative responses. To evaluate the final scores qualitatively, the scores above half (more than 50%) were considered high quality.

### Data extraction

Data were collected as follows: reference, location, type of study, sample size, age, duration of the prone position, proning protocol, timing of measurement, and respiratory parameters.

### Unification of units

All respiratory parameters converted to mmHg. For conversion of respiratory parameters to get from SI units (KPa) to mmHg was multiplied by 7.501.

### Statistical analysis

All analyses were conducted with Stata software version 14.0 (College Station, Texas). For each study, the mean and standard deviation (SD) of respiratory parameters in the prone position and supine position was extracted and if Median and IQR was reported; we changed it to mean with [(min + max + 2*Median)/4] or [(med + q1 + q3)/3] and SD with [IQR/1.35]. Then mean difference (MD) of respiratory parameters for each study was calculated by mean1 minus to mean 2. Due to different studies design (Before–After or Quasi-Experimental design), in the before–after design, we calculated the change score MD (mean after prone position minus mean before prone position), and in Quasi-Experimental design, we calculated MD (mean in supine position minus mean in prone position). Then Standard deviation in Before–After design and Quasi-Experimental design was calculated based on formulas (1) and (2):

1 $$ SD_{{{\text{change}}\;{\text{score}}}}  = \sqrt {SD_{{{\text{before}}}} ^{2}  + SD_{{{\text{after}}}} ^{2}  - \left( {2 \times r \times SD_{{{\text{before}}}}  \times SD_{{{\text{after}}}} } \right)} $$

where SD_before_, SD_after_, and Corr is the standard deviation in before prone position, standard deviation after prone position, and correlation coefficient between before and after

2 $$ {\text{SD}}_{{{\text{pooled}}}}  = \sqrt {\frac{{\left( {{\text{n}}_{1}  - 1} \right){\text{SD}}_{{{\text{prone}}\;{\text{position}}}}^{2}  + \left( {{\text{n}}_{2}  - 1} \right){\text{SD}}_{{{\text{supine}}\;{\text{position}}}}^{2} }}{{{\text{n}}_{1}  + {\text{n}}_{2}  - 2}}} $$

where SD_prone position_, SD_supine position_, n_1_, and n_2_ is the standard deviation in prone position group, the standard deviation in supine position group, the sample size in the prone position and supine position groups. Then pooled MD (PMD) was calculated by the “Metan” command^23,24^. Heterogeneity was determined using Cochran’s Q test of heterogeneity, and the I^2^ index was used to quantify heterogeneity. In accordance with the Higgins classification approach, I^2^ values above 0.7 were considered as high heterogeneity. To estimate the PMD for respiratory parameters and subgroup analysis (study design and ventilation), the fixed-effect model was used, and when the heterogeneity was greater than 0.7, the random-effects model was used. The meta-regression analysis was used to examine the effect of study design, sample size, BMI, age and prone position (PP) duration as factors affecting heterogeneity among studies. The “Meta bias” command was used to check for publication bias, and if there was any publication bias, the PMD was adjusted with the “Metatrim” command using the trim-and-fill method. In all analyses, a significance level of 0.05 was considered.

## Result

Overall, 1970 studies were found through databases. After excluding redundant papers, 855 studies remained. After reading abstracts, 775 studies were excluded from the list. Then, the full text of the remaining 80 studies was reviewed, and 52 studies were excluded. Finally, 28 studies included in qualitative analysis and 26 studies with a total sample size of 1272 participants were included in the quantitative analysis. The flowchart of this selection process is shown in Fig. 1. Studies were published during 2020–2021, most studies were done in the UK, China, and Spain with three studies and range participants age were 17–83 years old (Tables 1 and 2). Supplementary 2 shows risk of bias assessment for included studies. All studies were high quality (more than 50% scores).

**Table 1:** Overview of all included studies in systematic review.

ID	Author (Ref.)	Recruitment period	Country	Study type	Population/SS	Gender/age (year) Mean (SD)/median (IQR)/range	Duration of PP	Proning protocol/timing of measurement (hour)
1	Abou Arab^25^	1 March to 30 April, 2020	France	Before–after	Mechanically ventilated COVID-19 T: 25	Male/female	At least one 16-h PP session	H_0_: Before PP H_1_: At the end of the first 16-h PP session
2	Coppo^5^	20 March to 9 April, 2020	Italy	Before–after	COVID-19-related pneumonia T: 56	Male: 44 Female: 12 Age: 18–75	At least 3 h	H_0_: before PP H_1_: 10 min after pronation H_2_: 1 h after returning to the supine position
3	Ferrando^26^	12 March to 9 June, 2020	Spain and Andorra	Quasi-experimental	COVID-19 patients with ARF Case: 55 Control: 144 T: 199	Male/female	16 h/day during 3 consecutive day	Case: HFNO + awake PP Control: only receive HFNO H_0_: Before PP H_1_: After PP
4	Caputo^27^	1 March to 1 April, 2020	USA	Before–after	COVID-19 Hypoxemia (SpO_2_ < 90%) T: 50	Male/female Age: 59 (50–68)	5 min	Awake self proning with supplemental oxygen H_0_: At triage H_1_: With Supplemental oxygenation H_2_: After 5 min of proning
5	Ni^4^	31 January to 15 February, 2020	China	Quasi-experimental	COVID-19 Case: 17 Control: 35 T: 52	Male/female Age: 62 (12)	At least 4 h/day for 10 days	G_1_: Standard care G_2_: Position care (prone or lateral)
6	Elharrar^28^	27 March to 8 April, 2020	France	Before–after	COVID-19 T: 24	Male/female Age: 66.1 (10.2)	PP subgroup: Between less than 1 h to more than 3 h based on tolerability < 1 h (n: 4) 1 to < 3 h (n: 5) ≥ 3 h (n: 15)	H_0_:Before PP H_1_: During PP H_2_: 6 to 12 h after resupination
7	Retucci^29^	March and April 2020	Italy	Quasi-experimental	COVID-19 with spontaneous breathing/T: 26	Male/female Age: ≥ 18	1 h session/39 sessions: Case:12 prone session Control: 27lateral session	Prone (case) and lateral position (control) in Noninvasive Helmet CPAP Treatment H_0_: Before intervention H_1_: During intervention H_2_: 45 min after resupination
8	Mittermaier^30^	15 March to 11 April, 2020	Germany	Quasi-experimental	Mechanically ventilated COVID-19 T: 15	Male/female Age: 26–81	15 ± 2.5 h for 6.2 days	G_1_: Intubation G_2_: PEEP G_3_: PP
9	Taboada^31^	31 March to 11 April, 2020	Spain	Before–after	COVID-19 T: 29	Male/female Age: 64 (12)	1 h	H_0_: Before PP H_1_: During PP H_2_: After PP
10	Taboada^17^	15 March to 15 April, 2020	Spain	Before–after	COVID-19 T: 50	Male/female Age: 63 (53–71)	30–60 min	H_0_: Supine position H_1_: PP H_2_: Resupination
11	Zang^42^	1 February to 30 April, 2020	China	Before–after	COVID-19 Case: 23 Control: 37 T: 60	Male/female	Median: 9 h (8–22)	H_0_: Before PP H_1_: 10 min after PP H_2_: 30 min after PP
12	Dong^19^	5 February to 29 February, 2020	China	Before–after	COVID-19 T: 25	Male/female Age: 54.4 (16.1)	PP session > 4 h/day Mean (SD): 4.9 (3.1) h	Lateral positioning if PP not tolerated H_0_: Before PP H_1_: After sessions of PP
13	Shelhamer^11^	25 March to 2 May, 2020	USA	Quasi-experimental	Mechanically ventilated patients with moderate to severe ARDS due to COVID-19 Case: 62 Control: 199 T: 261	Male/female Age: 64 (55–73)	At least 16 h	Case: Prone Control: Not prone
14	Thompson^33^	6 April 6 to 14 April, 2020	USA	Before–after	COVID-19 with severe hypoxemic respiratory failure T: 25	Male/female	At least 1 awake session of the prone position lasting longer than 1 h	H_0_ : Supine position H_1_:1 h after initiation of PP
15	Tu^34^	1 February to 10 March, 2020	China	Before–after	COVID-19 T: 9	Male/female Age: 51 (11)	Median of 5 (IQR: 3–8) procedures per subject (twice daily). The median duration was 2 (IQR: 1–4) h	PP in HFNC H_0_:before PP H_1_: after PP
16	Weiss^16^	18 Marchto 31 March, 2020	USA	Before–after	Mechanically ventilated patients with COVID-19 T: 42	Male/female Age: 58.5 (51.8–69.3)	Several sessions lasting for 16 h	First PP session H_0_: Pre-prone (in 1 h) H_1_: Post-prone (in 2 h) H_2_: Post-prone (4 h after) H_3_: Pre-supine (0.5–2 h before) H_4_: Post-supine (0.5–2 h after)
17	Winearls^35^	8 April to 31 May, 2020	UK	Before–after	COVID-19 T: 24	Male/female Age: 62 (13)	Mean duration of PP was 8 ± 5 h for a mean of 10 ± 5 days	PP combined with CPAP H_0_: Prior to CPAP initiation H_1_: On CPAP prior to PP H_2_: During PP on CPAP (15 min after PP initiation) H_3_: 1 h after PP while on CPAP
18	Khullar^8^	March and May 2020	USA	Before–after	Mechanically ventilated SARS-CoV-2-positive adults/ Living (n = 6) deceased (n = 17) T: 23	Male/female Age: 57 (25–75)	≥ 16 h, ≥ 1 day	H_0_: Before PP H_1_: Post proning H_2_: 48 h after PP
19	Sharp^36^	12 March to 20 April, 2020	UK	Quasi-experimental	Mechanically ventilated COVID-19 pneumonia T: 12	Male/female Age: 30–76	Two or more full proning cycles	H_0_:Supine position H_1_: Prone position
20	Wendt^37^	30 March to 4 April, 2020	USA	Before–after	Spontaneously breathing COVID-19 with hypoxic respiratory distress T: 31	Male/female Age: 31(5)	At least 2 h	H_0_: Room air H_1_: Before PP with supplemental O_2_ H_2_: With PP
21	Berril^12^	23 March to 7 May, 2020	UK	Before–after	Mechanically ventilated COVID-19 T: 34	Female: 34 Age: (Med ± SD) 58.5 ± 11.1	The average duration was 16.5 ± 2.7 h/patient Proning done on average for 4 ± 2.4 separate sessions Total session: 131	H_0_: Before PP H_1_: After 3 h of PP
22	Burton-Papp^38^	4 March to 11 May, 2020	UK	Before–after	COVID-19 G_1_: 13 G_2_: 7 T: 20	Male/female Age: 53.4 (8.3)	5 prone cycles (each cycle lasted up to 3 h)	PP inconjunction with NIV G_1_: Only NIV G_2_: NIV and IMV T: All NIV and PP
23	Carsetti^1^	NR	Italy	Before–after	Mechanically ventilated SARS-CoV-2 T:10	Male: 10 Age: 58 (50–64)	Standard duration: 16 h Prolonged duration: 36 h	H_0_: Before pronation H_1_:During pronation H_2_: Resupination
24	Jagan^39^	24 March to 5 May, 2020	Grand Island	Quasi-experimental	COVID-19 G_1_: 40 G_2_: 65 T: 105	Male/female Age: G_1_: 56.0 (14.4) G_2_: 65.8 (16.3)	1 h	G_1_: Proning G_2_: Not proning
25	Padrao^9^	1 March to 30 April, 2020	Brazil	Quasi-experimental	COVID-19 hypoxemic respiratory failure/case: 57 Control: 109 T: 166	Male/female Age: 58.1 (14.1)	Between 30 min and 4 h	Case: PP Control: Not PP H_0_: Before PP H_1_: After PP
26	Sartini^32^	April 2, 2020	Italy	Before–after	Hypoxemic COVID-19 (SpO_2_ < 94%) T: 15	Male/female Age: 59 (6.5)	Median 3 h (IQR, 1–6 h)	PP for NIV patients H_0_: Before NIV H_1_: During NIV in pronation (60 min after start) H_2_: 60 min after NIV end
27	San^40^	1 April to 31 May, 2020	Turkey	Before–after	COVID-19 pneumonia (SpO_2_ < 93%) T: 21	Male/female Age: 71 (60–76.5)	G_1_ = 15 min or below (N = 7) G_2_ = Above 1 min (N = 14)	PP on the ambulance stretcher H_0_: Before transport H_1_: After transport
28	Solverson^41^	1 April to 25 May, 2020	Canada	Before–after	Non-intubated COVID-19 patients T: 17	Male/female Age: Median (range) 53 (34–81)	The median number of daily prone positioning sessions was 2 (1–6) with a duration of 75 (30–480) min for the first session G_1_ = < 75 min (n = 8) G_2_ = ≥ 75 min (n = 9)	H_0_: Supine position H_1_: Prone position H_2_: Resupination

*SS* sample size, *PP* prone position, *H* hour, *min* minutes, *G* group, *T* total, *O*_*2*_ oxygen, *NIV* non invasive ventilation, *IMV* intermittent mandatory ventilation, *HFNO* high flow nasal oxygen, *PEEP* positive end expiratory pressure, *CPAP* continuous positive airway pressure, *SD* standard deviation, *IQR* interquartile range.

**Table 2:** Respiratory parameters, intubation rate, and death rate in COVID-19 patients.

ID	Author	SPO_2_ (Sao_2_) (%) Mean (SD)/median (IQR)	PaO_2_/FIO_2_ ratio or SPO_2_/FIO_2_ ratio Mean (SD)/median (IQR)	PaCO_2_ (mmHg) Mean (SD)/median (IQR)	PaO_2_ (mmHg) Mean (SD)/median (IQR)	RR Mean (SD)/median (IQR)	Other variables
1	Abou-Arab	NR	H_0_: 91 (78–137) H_1_: 124 (97–149)	H_0_: 49 (42–51) H_1_: 49 (44–57)	NR	NR	NR
2	Coppo	H_0_: 97.2 (2·8) H_1_: 98·2 (2·2) H_2_: 97·1(1·9)	H_0_: 180.5 (76·6) H_1_: 285.5 (112·9) H_2_: 192·9 (100·9)	H_0_: 35.3 (4·9) H_1_: 35.6 (4·5) H_2_: 35.5 (4·4)	H_0_: 117.1 (47·4) H_1_: 200.4 (110·9) H_2_: 121·4 (69·6)	H_0_: 24.5 (5·5) H_1_: 24 (6·9) H_2_: 23·9 (6·3)	Intubation rate 18/56
3	Ferrando	H_0_ Case: 90.4 Control: 90.4 H_1_ Case : 87.6 Control: 88.8	H_0_ Case: 148.2 Control: 123.9 H_1_ Case: 113.8 Control: 109.7	H_0_ Case: 34.0 Control: 34.7 H_1_ Case: 42.4 Control: 44.8	NR	H_0_ Case: 25.5 Control: 25.7 H_1_ Case Minimum: 20.8 Maximum: 27.7 Control Minimum: 19.7 Maximum: 27.1	Intubation rate Hazard ratio (95% CI); 1.002 (0.531, 1.890) 28-day mortality rate Hazard ratio (95% CI); 2.411 (0.556, 10.442)
4	Caputo	H_0_: 80 H_1_: 84 H_2_: 94	NR	NR	NR	NR	Intubation rate 13/50
5	Ni	NR	H_0_ G_1_: 128 (60) G_2_: 142 (54) T: 133 (58) Spo_2_/Fio_2_ 409 (95% CI 86–733)	NR	NR	H_0_ G_1_: 26 ( 5) G_2_: 23 (4) T: 25 (5)	NR
6	Elharrar	NR	NR	Total H_0_: 34.1 (5.3) H_1_: 32.8 (4.5) H_2_: 32.3 (5.1)	Total H_0_: 72.8 (14.2) H_1_: 91 (27.3) H_2_: 77.6 (11.5)	Total H_0_: 18 (2.7)	Intubation rate 5/24
7	Retucci	Total H_0_: 96 (95–98) H_1_: 98 (97–98) H_2_: 97 (95–98) Case H_0_: 95 (93.5–96.0) H_1_: 98 (98–99) H_2_: 96 (95–98) Control H_0_: 97 (96–98) H_1_: 98 (96–98) H_2_: 97 (96–98)	Total H_0_: 182.9 (43.0) H_1_: 220.0 (64.5) H_2_: 179.3 (43.9) Case H_0_: 168.7 (46.2) H_1_: 227.7 (90.3) H_2_: 166.9 (45.3) Control H_0_: 189.7 (40.6) H_1_: 216.2 (49.6) H_2_: 185.0 (43.0	Total H_0_: 38 (35–40) H_1_: 38 (35–39) H_2_: 38 (35–40) Case H_0_: 39 (35.5–40.5) H_1_: 38 (34.5–41.0) H_2_: 37 (35–41) Control H_0_: 38 (34–39) H_1_: 37 (35–39) H_2_: 38 (35–40)	Total H_0_: 86.9 (15.1) H_1_: 104.5 (25.0) H_2_: 85.4 (13.4) Case H_0_: 83.6 (14.2) H_1_: 112.3 (32.3) H_2_: 85.6 (11.5) Control H_0_: 88.4 (15.5) H_1_: 100.8 (20.4) H_2_: 85.8 (14.5)	Total H_0_: 23.7 (4.7) H_1_: 23.1 (4.5) H_2_: 23.6 (4.7) Case H_0_: 23.5 (6.3) H_1_: 21.3 (5.0) H_2_: 22.9 (6.0) Control H_0_: 23.8 (.9) H_1_: 23.9 (4.0) H_2_: 24.0 (4.1)	Intubation rate 7/26 (26.9%) Death rate 2/26 (7.7%)
8	Mittermaier	NR	H_0_ G_1_: 84.3(28) G_2_: 80^a^ G_3_: 140^a^ H_1_ G_1_: 210.7 (86.6) G_2_: 197.9 (43.0) G_3_: 190^a^	H_0_ G_1_: 35.9(7) H_1_ G_2_: 52.4 (9.7)	H_0_ NR H_1_ G_2_: 79.5(7.8)	H_0_ G_1_: 31 (2.6) G2: 16 (2.6) H_1_ G_2_: 15.7 (2.8)	Death rate G_1_ = 40% G_2_ = 42.9% G_3_ = 55.6%
9	Taboada	H_0_: 93.6 (2.3) H_1_: 95.8 (2.1) H_2_: 95.4 (2.7)	H_0_: 196 (68) H_2_: 242 (107)	NR	H0: 75^a^ H1: 80^a^	NR	Death rate 2/29 (7%)
10	Taboada	NR	NR	NR	NR	NR	Death rate 4%
11	Zang	Case H_0_: 91.09 (1.54) H_1_: 95.30 (1.72) H_2_: 95.48 (1.73)	NR	NR	NR	Case H_0_: 28.22 (3.06) H_1_: 27.78 (2.75) H_2_: 24.87 (1.84)	Death rate Case: 10/23 (43.5%) Control: 28/37 (75.7%)
12	Dong	NR	H_0_: 194 (164–252) H_1_: 348 (288–390)	NR	NR	H_0_: 28.4 (3.5) H_1_: 21.3 (1.3)	Death rate 0/25
13	Shelhamer	NR	PaO_2_/FIO_2_ Case 0.10 (0.04, 0.17) + 11% improvement SPO_2_/FiO_2_ − 0.28 (0.63, 0.08) + 24% improvement	NR	NR	NR	Death rate Case: 48 (77.4%) Control: 167 (83.9%)
14	Thompson	H_0_: 65–95%^a^ H_1_: 90–100%* + (1–34%) [median [SE], 7% [1.2%]; 95% CI 4.6–9.4%)	NR	NR	NR	NR	Intubation rate 12/25 (48%) Death rate 3/25 (10%)
15	Tu	H_0_: 90 (2) H_1_: 96 (3)	NR	H_0_: 47 (7) H_1_: 39 (5)	H_0_: 69 (10) H_1_: 108 (14)	NR	Intubation rate 2/9
16	Weiss	H_0_: 96 (93–99.0) H_1_: 97.5(95–99) H_2_:97 (95.0–99.0) H_3_:98 (96–99.0) H_4_: 96.5 (94.0- 99.0)	(KPa) H_0_: 17.5 (11.6–19.2) H_1_: 27.7 (19.5–35.7) H_2_: NR H_3_: NR H_4_: 26.1 (17.9–33.1)	(KPa) H_0_: 7.2 (5.7–7.9) H_1_: 6.8 (6.0–7.7) H_2_: NR H_3_: NR H_4_: 6.3 (5.5–6.8)	(KPa) H_0_: 11.8 (9.3–14.2) H_1_: 14.5 (10.2–20.4) H_2_: NR H_3_: NR H_4_: 13.5 (10.3–17.3)	NR	Death rate 11/42
17	Winearls	H_0_: 94 (3) H_1_: 95 (2) H_2_: 96 (2) H_3_: 96 (2)	H_0_: 143 (73) H_1_: 201 (70) H_2_: 252 (87) H_3_: 234 (107)	NR	NR	H_0_: 27 (6) H_1_: 25 (6) H_2_: 24 (6) H_3_: 25 (6)	Death rate 4/24
18	Khullar	NR	Living H_0_: 86.5^a^ H_1_: 180^a^ H_2_: 115^a^ Deceased H_0_: 84.2^a^ H_1_: 210^a^ H_2_: 107^a^ Total H_0_: 84.8^a^ H_1_: 202^a^ H_2_: 109^a^	NR	Living H_0_: 86.5^a^ H_1_: 138^a^ H_2_: 68.2^a^ Deceased H_0_: 77.1^a^ H_1_:185^a^ H_2_: 82.8^a^ Total H_0_: 79.5^a^ H_1_: 173^a^ H_2_: 78.8^a^	H_0_: 27.2 H_1_: 23.6	NR
19	Sharp	NR	H_0_: 88.95 (19.34) H_1_: 110.18 (28.11)	NR	NR	NR	30 day mortality rate 9/12
20	Wendt	H_0_: 83% (IQR: 75–86%) H_1_: 90% (IQR: 89–93%) H_2_: 96% (IQR: 94–98%)	NR	NR	NR	H_1_:31 (SD = 9) H_2_: 26 (SD = 8)	Intubation rate 14/31 Death rate 8/31
21	Berril	NR	(N: 89 session) H_0_: 99.8 (37.5) H_1_: 151.9 (58.9)	H_0_: 47.3 ( 8.9)	NR	H_0_:18 (4.2)	Death rate 17/34 (50%)
22	Burton-Papp	NR	Δ PaO_2_/FiO_2_ G_1_: + 40.8 (95% CI 28.8–52.7) G_2_: + 5.06 (95% CI − 9.5 to 19.75) T: + 28.7 mmHg [95% CI 18.7–38.6]	NR	NR	Δ RR G_1_: − 1.27 (95% CI − 2.4 to − 0.1) G_2_: − 0.09 ± 6.45 (95% CI − 2.3 to 2.1) T: − 0.98 [95% CI − 2 to 0.04]	Intubation rate 7/20 (35%) Death rate 0%
23	Carsetti	NR	Standard pronation H_1_ vs. H_0_ H_2_ vs. H_1_ Prolonged pronation H_1_ vs. H_0_ H_2_ vs. H_0_	NR	NR	NR	NR
24	Jagan	NR	(95% CI 29.6 lower to 10.8 higher)	NR	NR	NR	Death rate G_1_: 0 G_2_: 24.6% Intubation rate G_1_: 10% G_2_: 27.7%
25	Padrao	Case H_0_: 92 (88–93) H_1_: 94 (92–96)	Case H_0_: 196 (128- 254) H_1_: 224 (159–307)	NR	NR	Case H_0_: 34 (30–38) H_1_: 29 (26–32)	Intubation rate Case: 33/57 (58%) Control: 53/109 (49%) Death rate Case: 6 (11%) Control: 22 (20%)
26	Sartini	H_0_: 93.5^a^ H_1_: 118.6^a^ H_2_: 95.3^a^	H_0_: 91^a^ H_1_: 129^a^ H_2_: 90.2^a^	NR	NR	H_0_: 26.6^a^ H_1_: 23.5^a^ H_2_: 23.1^a^	Intubation rate 1/15 Death rate 1/15
27	san	G_1_ H_0_: 90.1 (82.3–92.5) H_1_: 91.0 (89.1–93.4) G_2_ H_0_: 87.9 (5.6) H_1_:94.1 (3.5) Total H_0_: 89.6 (83.6–91.8) H_1_: 92.8 (89.9–97.1)	NR	G_1_ H_0_: 38.5 (29.7–51.2) H_1_: 36.7 (34.1–47.1) G_2_ H_0_: 37.4 (33.6–41.0) H_1_: 35.3 (31.3–43.9) Total H_0_: 37.8 (32.7–44.5) H_1_: 35.6 (33.2–44.7)	G_1_ H_0_: 64.5 (18.2) H_1_: 67.9 (13.4) G_2_ H_0_: 53.3 (45.4–67.4) H_1_: 71.0 (63.1–104.1) Total H_0_: 53.5 (46.1 71.0) H_1_: 70.0 (60.7–88.1)	NR	NR
28	Solverson	G_1_ H_0_: 91 (87–95) H_1_:98 (94–100) G_2_ H_0_: 91 (84–95) H_1_: 96 (92–99) Total H_0_: 91 (84–95) H_1_: 98 (92–100)	G_1_ H_0_: 138 (97–198) H_1_: 155 (106–248) G_2_ H_0_: 152 (97–233) H_1_: 165 (106–240) Total H_0_: 152 (97–233) H_1_: 165 (106–248)	NR	NR	G_1_ H_0_: 30 (24–38) H_1_: 20 (15–33) G_2_ H_0_: 26 (18–35) H_1_: 24 (16–32) Total H_0_: 28 (18–38) H_1_: 22 (15–33)	Intubation rate 7/17 Death rate 2/17

*H* hour, *Spo*_*2*_ pulse oximeter oxygen saturation, *Sao*_*2*_ oxygen saturation (arterial blood), *Paco*_*2*_ partial pressure of carbon dioxide, *Pao*_*2*_ partial pressure of oxygen, *FIO*_*2*_ fractional inspiratory oxygen, *RR* respiratory rate, *SD* standard deviation, *IQR* interquartile range, *mmHg* millimeter of mercury, *CI* confidence interval, *SE* standard error, *SHR* subdistibution hazard ratio, *SS* sample size, *NR* not reported.

^a^Data extracted from figures and charts.

### Pooled mean difference of respiratory parameters in total and based on subgroups

**Figure 2:**
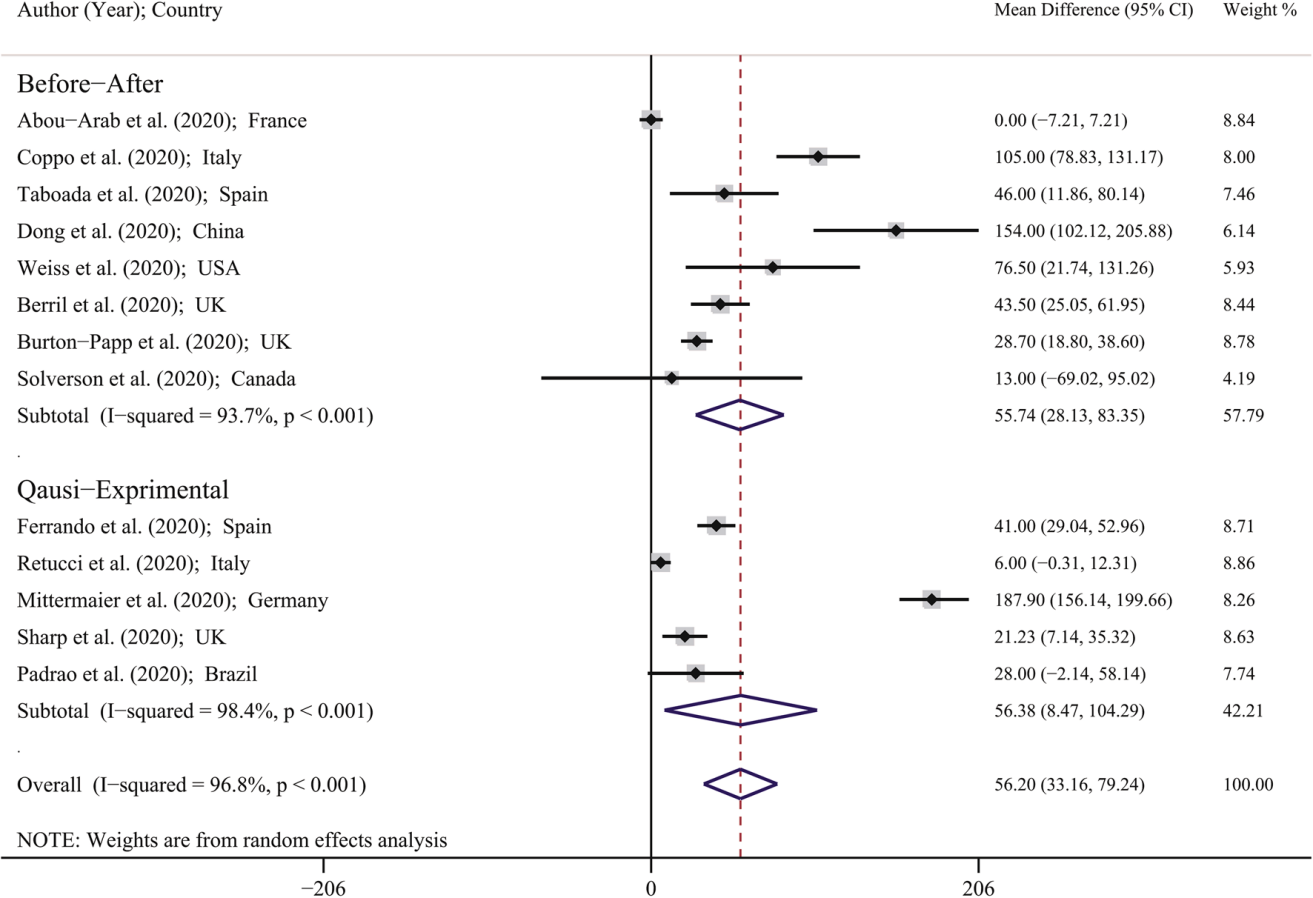
Forest plot for mean difference (MD) of PaO_2_/FIO_2_ Ratio (mmHg) based on random effects model. The midpoint of each line segment shows the MD, the length of the line segment indicates the 95% confidence interval in each study, and the diamond mark illustrates the pooled MD.

Figure 2 showed the forest plot for MD of PaO_2_/FIO_2_ Ratio in included studies. The minimum and maximum reported MD of PaO_2_/FIO_2_ reported by Abou-Arab et al. (MD: 0.00; 95% CI 7.21–7.21 mmHg) in France^25^ and by Mittermaier et al. (MD: 187.90; 95% CI 156.14–199.66 mmHg) in Germany^30^. Based on Fig. 2 using the random-effects model approach; the PMD in the study with before–after design, quasi-experimental design and in total was 55.74 (95% CI 28.13–83.35) mmHg, 56.38 (95% CI 8.47–104.29) mmHg, and 56.20 (95% CI 33.16–79.24) mmHg; respectively. This means that in general, the prone position in COVID-19 patients leads to significant improvement of PaO_2_/FIO_2_ Ratio, so that in before–after design, quasi-experimental design, and in total, the mean of PaO_2_/FIO_2_ Ratio significantly increased 55.74, 56.38, and 56.20 mmHg; respectively.

**Figure 3:**
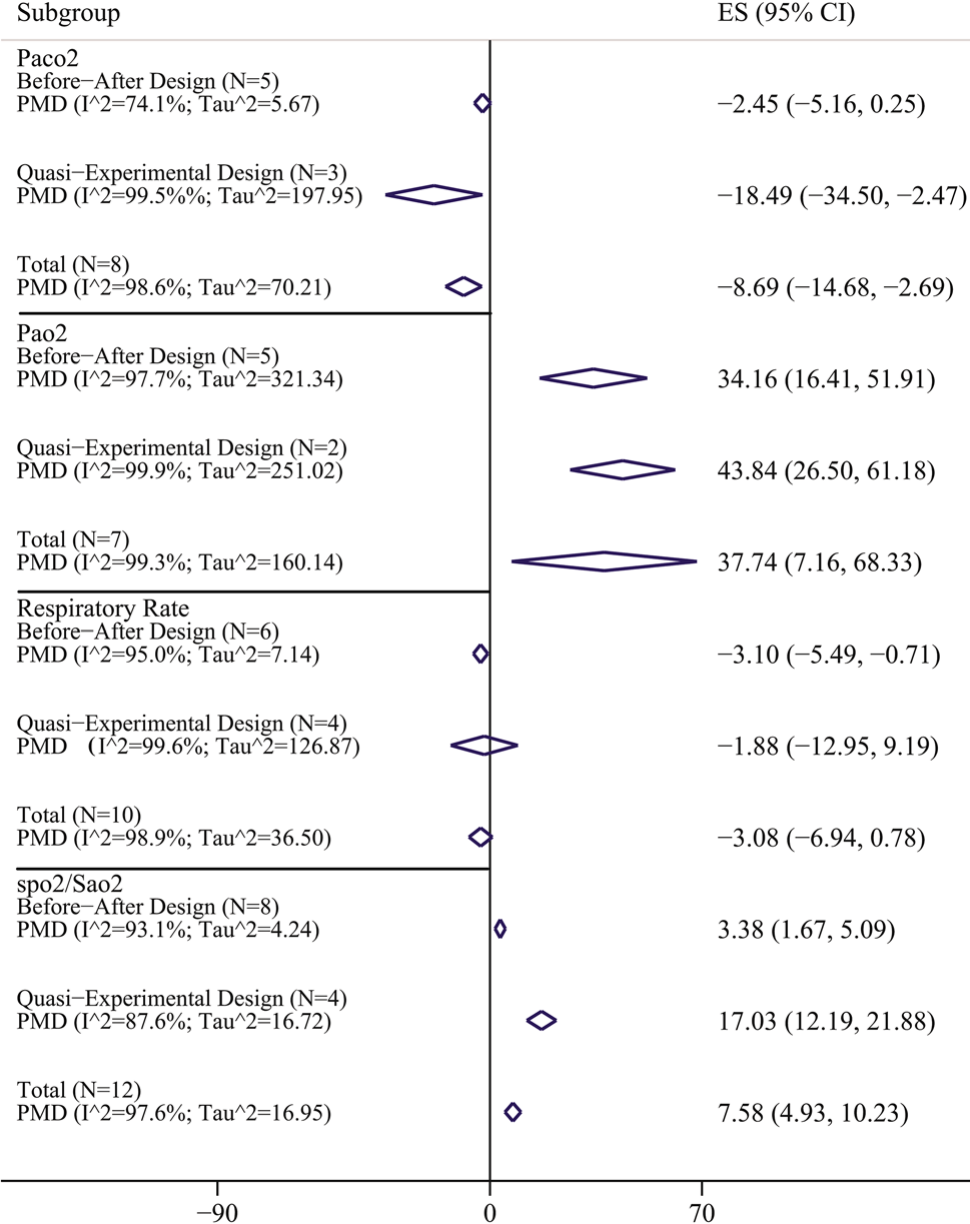
Pooled mean difference and 95% confidence interval of respiratory parameters based on the random effects model in total and in different study design. The diamond mark illustrates the pooled estimate.

**Figure 4:**
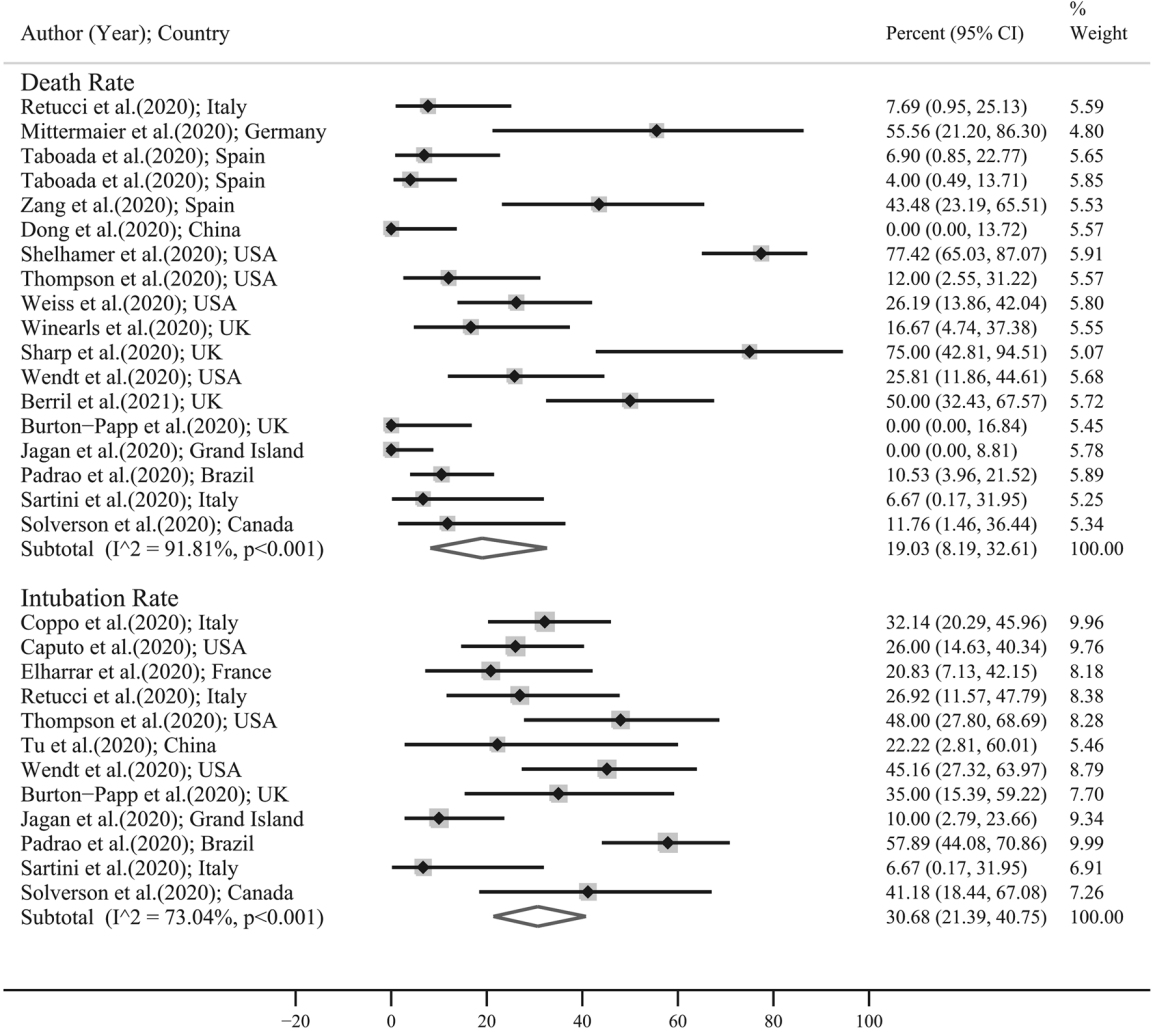
Forest plot for death rate and intubation rate in included studies. The diamond mark illustrates the pooled estimate and length of diamond indicates 95% confidence interval.

Figure 3 and Table 3 showed the PMD of other respiratory parameters in included studies. The PMD of SPO_2_ (Sao_2_) in the study with before–after design, quasi-experimental design, and in total was 3.38 (95% CI 1.68–5.09), 17.03 (95% CI 12.19–21.88), and 7.58 (95% CI 4.93–10.23); respectively. This means that the prone position in COVID-19 patients leads to significant improvement corresponding to Spo_2_ (Sao_2_). Also the PMD of Paco_2_ in COVID-19 patients was significantly decreased in quasi-experimental design (PMD: − 18.49; 95% CI − 34.50 to − 2.47 mmHg) and in total (PMD: − 8.69; 95% CI − 14.69 to − 2.69 mmHg). No significant change was observed for PMD of PaCo_2_ in the before–after design. The PMD of other respiratory parameters showed in Table 3 and Fig. 3. It should be noted that prone position leads to improvement of PaO_2_ but does not have any effects on the respiratory rate in general, especially in the quasi-experimental design. The pooled estimate and 95% CI for death rate and intubation rate were 19.03 (8.19–32.61) and 30.68 (21.39–40.75); respectively (Fig. 4).

**Table 3:** Result of meta-analysis for calculation of pooled mean difference of respiratory parameters; publication bias and fill and trim method.

Variables	Subgroup	Meta-analysis	Heterogeneity		Egger's test for publication bias		Fill-and-trim
		PMD (95% CI)	I^2^ (%)	Tau^2^	Coefficient (95% CI)	P-value	PMD (95% CI)
PaO_2_/FIO_2_ ratio	Before–after design (N = 8)	55.74 (28.13–83.35)	93.7	121.01	5.63 (0.91–10.35)	0.024	57.41 (32.19–81.01)
	Quasi-experimental design (N = 4)	56.38 (8.47–104.29)	98.4	141.02			
	Total (N = 12)	56.20 (33.16–79.24)	96.8	99.04			
Spo_2_ (Sao_2_)	Before–after design (N = 8)	3.38 (1.68–5.09)	93.1	4.24	− 10.02 (− 25.04 to 5.01)	0.168	–
	Quasi-experimental design (N = 4)	17.03 (12.19–21.88)	87.6	16.72			
	Total (N = 12)	7.58 (4.93–10.23)	97.6	16.95			
Paco_2_	Before–after design (N = 5)	− 2.45 (− 5.15 to 0.25)	74.1	5.67	− 3.89 (− 16.71 to 8.94)	0.486	–
	Quasi-experimental design (N = 3)	− 18.49 (− 34.50 to − 2.47)	99.5	197.95			
	Total (N = 8)	− 8.69 (− 14.69 to − 2.69)	98.6	70.21			
Pao_2_	Before–after design (N = 5)	34.16 (16.41–51.91)	87.7	321.34	2.12 (− 18.16 to 22.40)	0.799	–
	Quasi-experimental design (N = 2)	43.84 (26.03–61.18)	99.9	251.02			
	Total (N = 7)	37.74 (7.16–68.33)	99.3	160.14			
RR	Before–after design (N = 6)	− 3.10 (− 5.49 to − 0.71)	95.0	7.14	1.52 (− 12.94 to 15.98)	0.815	–
	Quasi-experimental design (N = 4)	− 1.88 (− 12.95 to 9.19)	99.6	126.87			
	Total (N = 10)	− 3.08 (− 6.94 to 0.78)	98.9	36.50			

*CI* confidence interval, *N* number of study, *PMD* pooled mean difference, *Pao*_*2*_ partial pressure of oxygen, *FIO*_*2*_ fractional inspiratory oxygen, *Sao*_*2*_ oxygen saturation (arterial blood), *RR* respiratory rate.

**Figure 5:**
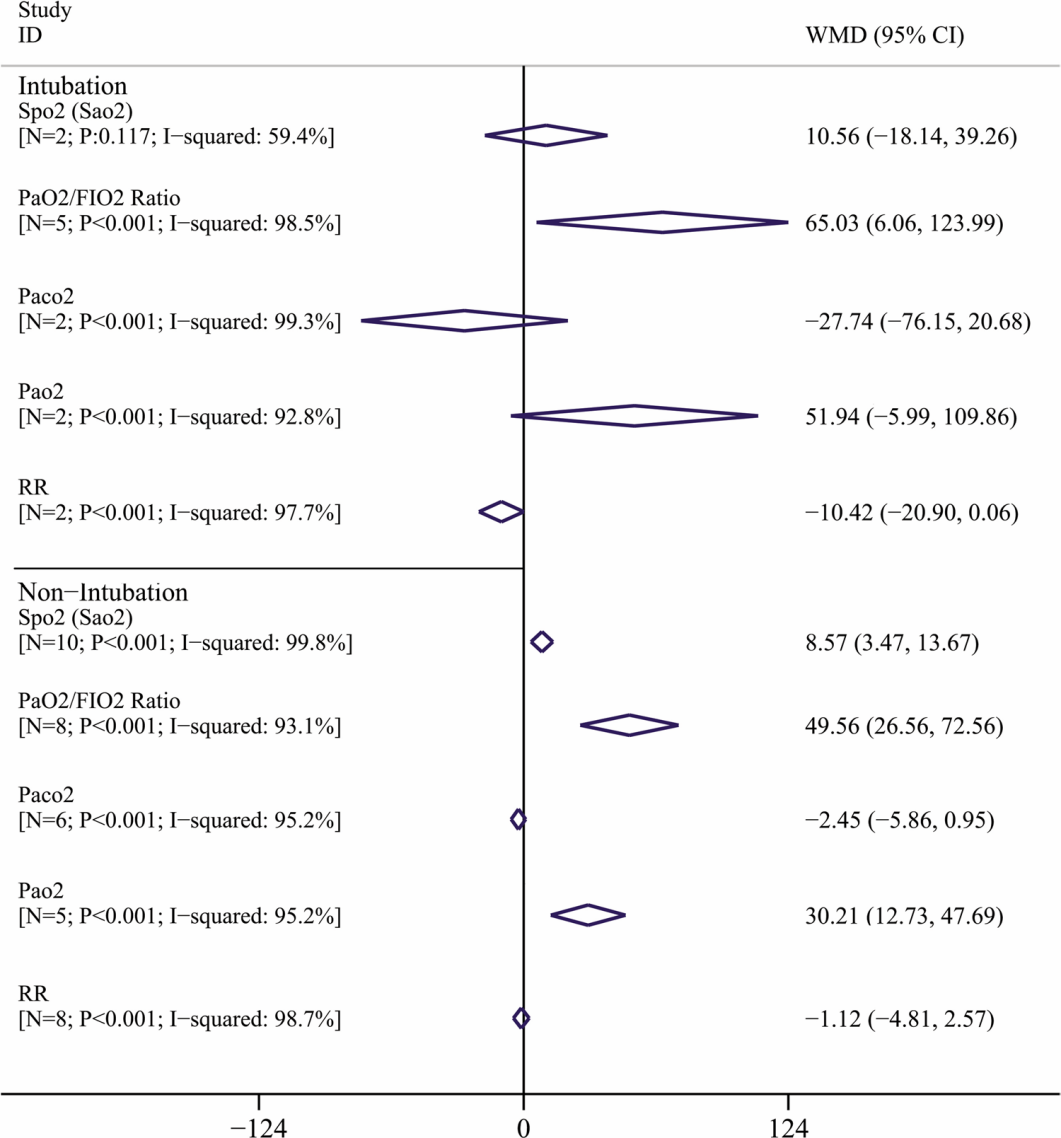
Pooled mean difference and 95% confidence interval of respiratory parameters based on the random effects model in different ventilation status. The diamond mark illustrates the pooled estimate.

Figure 5 showed PMD of respiratory parameters based on ventilation status. PMD of Spo_2_ (Sao_2_) in Intubation and Non-intubation subgroup was 10.56 (95% CI − 18.15 to 39.26) and 8.57 (95% CI 3.47–13.67); respectively. This means that the prone position in COVID-19 patients with non-intubation leads to significant improvement corresponding to Spo_2_ (Sao_2_) but Intubation have no effects on Spo_2_ (Sao_2_) improvement. Also PMD of PaO_2_/FIO_2_in Intubation and non-intubation subgroup was 65.03 (95% CI 6.06–123.99) and 49.56 (95% CI 26.56–72.56); respectively. This means that the prone position in COVID-19 patients leads to significant improvement of PaO_2_/FIO_2_ Ratio, but this value for Intubated patients was higher than non-intubated groups. Situation of other parameter was showed in Fig. 5.

### Publication bias

Based on Egger's test results, significant publication bias was observed for PaO_2_/FIO_2_ Ratio (Coefficient: 5.63; 95% CI 0.91–10.35; p: 0.024). Therefore, the fill- and trim-adjusted PaO_2_/FIO_2_ Ratio (PMD: 57.41, 95% CI 32.19–81.01 mmHg) was generated, which was not significantly different from the original PaO_2_/FIO_2_ Ratio (PMD: 56.20; 95% CI 33.16–79.24 mmHg). It means that the result of the meta-analysis was robust. No significant publication bias was observed for other respiratory parameters.

### Heterogeneity and meta-regression results

According to Cochran’s Q test of heterogeneity, there was significant heterogeneity among studies (p < 0.001). Except for PaCo_2_ in the before–after design, the heterogeneity amount was more than 85% based on the I^2^ index, which indicates high heterogeneity. Table 4 presents the results of the univariate meta-regression; there are significant associations between study, results with study design corresponding to SPO_2_ (Sao_2_) percent (Coefficient: 12.80; p < 0.001). No significant associations were observed for other respiratory parameters with sample size, study design, BMI, age and PP duration (Table 4).

**Table 4:** Results of the univariate meta-regression analysis on the heterogeneity of the determinants.

Variables	SPO_2_/Sao_2_ (%)	PaO_2_/FIO_2_ ratio (mmHg)	PaCo_2_ (mmHg)	PaO_2_ (mmHg)	RR (RPM)
**Sample size**					
Coefficient (95% CI)	0.04 (− 0.01 to 0.14)	− 0.15 (− 0.79 to 0.4789)	0.05 (− 0.23 to 0.33)	0.15 (− 2.01 to 2.31)	0.01 (− 0.11 to 0.15)
p-value	0.091	0.583	0.697	0.821	0.701
**Study design**					
Coefficient (95% CI)	12.80 (7.78 to 17.81)	− 1.22 (− 76.96 to 74.52)	− 15.71 (− 46.37 to 14.94)	8.80 (− 62.74 to 80.34)	2.12 (− 8.80 to 13.03)
p-value	< 0.001	0.972	0.256	0.765	0.667
**BMI**					
Coefficient (95% CI)	− 0.91 (− 5.66 to 3.83)	− 1.11 (− 32.82 to 30.59)	0.34 (− 22.74 to 23.43)	− 10.24 (− 50.94 to 30.47)	− 1.37 (− 55.51 to 52.76)
p-value	0.941	0.927	0.955	0.193	0.802
**Age**					
Coefficient (95% CI)	− 0.04 (− 1.35 to 1.26)	0.77 (− 11.46 to 13.01)	0.05 (− 2.97 to 3.07)	− 1.69 (− 6.90 to 3.52)	− 0.39 (− 1.42 to 2.20)
p-value	0.941	0.889	0.969	0.443	0.626
**PP duration**					
Coefficient (95% CI)	− 0.08 (− 1.22 to 1.05)	1.50 (− 4.36 to 7.36)	− 1.28 (− 3.94 to 1.38)	1.40 (− 4.94 to 7.73)	− 0.70 (− 1.52 to 0.13)
p-value	0.875	0.582	0.271	0.574	0.089

*CI* confidence interval, *mmHg* millimeter of mercury, *PMD* pooled mean difference, *PaO*_*2*_ partial arterial oxygen, *FIO*_*2*_ fractional inspiratory oxygen, *Sao*_*2*_ oxygen saturation (arterial blood), *RR* respiratory rate, *RPM* respiration per minute, *Study design* before–after design = 1; quasi-experimental design = 2.

**Figure 6:**
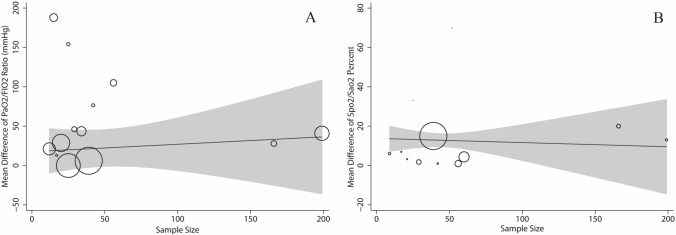
Association between sample size with mean difference (MD) of PaO_2_/FIO_2_ Ratio (mmHg) (**A**) and Spo_2_ (Sao_2_) (**B**) using meta-regression. Size of the circles indicates sample magnitude. There was no significant association between sample size with MD of PaO_2_/FIO_2_ Ratio and Spo_2_ (Sao_2_).

## Discussion

This systematic review analyzed the effects of prone position on respiratory parameters, intubation, and death rate. We found that prone position initiation leads to improved oxygenation parameters (PaO_2_/FiO_2_ ratio, SpO_2_, PaO_2_, and PaCO_2_) in patients with mild to severe respiratory failure due to confirmed COVID-19. However, the prone position did not change the respiratory rate in patients with hypoxemic respiratory failure suffering from COVID-19.

Most of the studies (18/28 studies) demonstrated significant improvement in PaO_2_/FiO_2_ ratio after prone positioning. Moreover, the improvement of SpO_2_ (SaO_2_) and PaO_2_ has been shown in 15 and 7 studies, respectively. Although the effect of prone position after resupination has declined in five studies^1,5,8,16,29^, early prone positioning should be considered as first-line therapy in ARDS patients^43^. Initiation of prone position in ARDS patients by reducing shunt, and V/Q mismatch, brings about an increase in the recruitment of non-aerated areas of the lungs, secretion clearance, improvement work of breathing (WOB) and oxygenation, and reduction of mortality compared with the supine position^44–46^. Prone position by enhancement in PaO_2_/FiO_2_ ratio not only leads to a decrease in the classification of respiratory failure but also prevents further complications due to ARDS, such as multi-organ failure (MOF)**,** which is the most common cause of mortality in this devastating condition^47^.

The efficacy of prone positioning may be affected by various protocols, such as different settings (ICU or emergency department), the timing of initiation (early or late), duration (prolonged or short sessions), positioning (prone position with or without lateral position), respiratory support in intubated or non-intubated patients (mechanical ventilation, NIV, nasal cannula, helmet, face mask) and the severity of ARDS^48^. Even though in this study PaO_2_/FiO_2_ ratio was significantly higher in the prone-positioning group with mild to severe ARDS, a further meta-analysis need to assess the impact of prone position in a different classification of ARDS with mild (PaO_2_/FiO_2_ = 201–300 mmHg), moderate (PaO_2_/FiO_2_ = 101–200 mmHg), and severe (PaO_2_/FiO_2_ < 100 mmHg) condition. In this systematic review, the prone position time varied from less than 1 to 16 h in a day. In eight studies, the prone positioning has been implemented for about 16 h a day. The prolonged prone positioning (no less than 10–12 h and ideally for 16–20 h) leads to improved oxygenation and a significant reduction in mortality in patients with severe ARDS. On the other hand, reducing the number of turning in patients with critical conditions can decrease the risk of more complications^48^. Although PaCO_2_ did not demonstrate a difference in five studies^5,16,25,29,40^, the PMD of PaCO_2_ in COVID-19 patients significantly decreased totally. The prone position by increasing the dorsal recruitment, PaCO2 clearance, and decreasing the dead space can also lead to better ventilation. Moreover, a higher PaCO_2_ clearance due to the prone position is related to a significant decrease in 28-day mortality^54^. In terms of respiratory rate, in few studies, the respiratory rate reduction was significant, but we found that respiratory rate did not change during the prone positioning in the overall analysis.

Our systematic review and meta-analysis demonstrated that prone positioning leads to a lower mortality rate in confirmed COVID-19 patients. Although in this systematic review and meta-analysis, many studies have assessed the impact of prone position on the short term (28 days) mortality, where they benefit from prone positioning protocols, the effect of prone positioning in the long-term (3 months or more) mortality is unclear. Therefore, further studies will be needed to demonstrate the relationship between prone positioning in COVID-19 patients and long-term mortality. Furthermore, this study confirmed that the improvement of oxygenation parameters due to the prone position might be associated with a lower intubation rate in COVID-19 patients.

## Conclusions

In our systematic review of 28 studies, prone positioning has been compared with supine positioning in hypoxic adult patients with COVID-19. We found prone position by optimizing lung recruitment, and the V/Q mismatch can improve oxygenation parameters such as PaO_2_/FIO_2_ Ratio, Spo_2_ (Sao_2_), PaO_2_, PaCO_2_. Nevertheless, the prone position did not change their respiratory rate. Moreover, the initiation of prone position might be associated with a lower mortality and intubation rate. Since most patients demonstrated improved oxygenation and lower mortality and intubation rate, we recommend the prone position in patients COVID-19.Similar to other studies, our research had some limitations. (1) Some studies did not report values of the respiratory parameters in different groups and just reported significantly parameter (like that p-value); which we have to exclude this studies from the quantitative analysis that this limitation was not be resolved even by data requesting from corresponding authors. We would like to perform the gender-specific estimation, but it was not possible due to insufficient data in the primary studies; (2) also we tend to estimate the pooled MD in different geographical regions or country-specific estimation based on available methods^50^, since the infrequent studies number, this estimation will not be robust.

## Supplementary Information

Supplementary Information.

## Abbreviations

PMD: Pooled mean difference
CI: Confidence interval
ARDS: Acute Respiratory Distress Syndrome
VILI: Ventilator-induced lung injury
PaO_2_: Pressure of arterial oxygen
FIO_2_: Fraction of inspired oxygen
WHO: World Health Organization
PP: Prone position
NIV: Non invasive ventilation
IMV: Intermittent mandatory ventilation
HFNO: High flow nasal oxygen
PEEP: Positive end expiratory pressure
CPAP: Continuous positive airway pressure
SD: Standard deviation
IQR: Interquartile range
