# Supplementary material for: Effect of prone position on respiratory parameters, intubation and death rate in COVID-19 patients: systematic review and meta-analysis
Source: Sci Rep. 2021 Jul 13;11:14407. doi: 10.1038/s41598-021-93739-y (PMC8277853; doi:10.1038/s41598-021-93739-y)
Supplement: Supplementary file 1 — Supplementary Information. [file 41598_2021_93739_MOESM1_ESM.docx]

**Supplementary 1**: Definitions and values of respiratory variables

| **Variable** | **definition** | **Normal value** |
| --- | --- | --- |
| PO_2_ | The partial pressure of oxygen (PO_2_) reflects the amount of oxygen gas dissolved in the blood. | 80-100 mmHg |
| PCO_2_ | Partial Pressure of Carbon Dioxide reflects the amount of CO_2_ dissolved in the blood. | 35-45 mmHg |
| SaO_2_ | Oxygen saturation measures the percentage of hemoglobin that is fully combined with oxygen. | 95-100% |
| SPO_2_ | Peripheral oxygen saturation measured by an oximetry. | 95% and above |
| PO_2_/FIO_2_ or SPO_2_/FIO_2_ | The ratio of partial pressure of arterial oxygen (PaO_2_) to the fraction of inspired oxygen (FiO_2_) which reflects hypoxemia in ARDS | Hypoxemia in ARDS:  Mild: 201–300 mmHg  Moderate: 101–200 mmHg severe: < 100 mmHg) |
| RR | Respiration frequency per minute | 12-20 breath/min |

**Supplementary 2**: Mixed Methods Appraisal Tool (MMAT)

| Quality | 5. During the study period, is the intervention administered (or exposure occurred) as intended? | 4. Are the confounders accounted for in the design and analysis? | 3. Are there complete outcome data? | 2. Are measurements appropriate regarding both the outcome and intervention (or exposure)? | 1. Are the participant’s representative of the target population? | ID |
| --- | --- | --- | --- | --- | --- | --- |
| High | C | C | Y | Y | Y | 1 |
| High | Y | Y | Y | Y | Y | 2 |
| High | Y | Y | N | Y | Y | 3 |
| High | Y | Y | Y | Y | Y | 4 |
| High | Y | Y | Y | Y | Y | 5 |
| High | Y | C | Y | Y | Y | 6 |
| High | Y | C | C | Y | Y | 7 |
| High | Y | Y | C | Y | Y | 8 |
| High | C | C | Y | Y | Y | 9 |
| High | C | C | Y | Y | Y | 10 |
| High | C | Y | Y | Y | Y | 11 |
| High | Y | Y | Y | Y | Y | 12 |
| High | Y | Y | Y | Y | Y | 13 |
| High | Y | C | Y | Y | Y | 14 |
| High | Y | N | C | Y | Y | 15 |
| High | Y | Y | Y | Y | Y | 16 |
| High | C | N | Y | Y | Y | 17 |
| High | Y | C | Y | Y | Y | 18 |
| High | Y | N | C | Y | Y | 19 |
| High | Y | C | Y | Y | Y | 20 |
| High | Y | C | C | Y | Y | 21 |
| High | Y | N | N | Y | Y | 22 |
| High | Y | N | N | Y | Y | 23 |
| High | Y | C | Y | Y | Y | 24 |
| High | Y | Y | Y | Y | Y | 25 |
| High | Y | C | Y | Y | Y | 26 |
| High | Y | C | Y | Y | Y | 27 |
| High | Y | C | Y | Y | Y | 28 |
| Y: Yes; N: No; C: Can’t tell | | | | | | |
